# Association between global longitudinal strain and clinical outcomes following COVID‐19 infection

**DOI:** 10.1002/clc.23788

**Published:** 2022-02-21

**Authors:** Teruhiko Imamura

**Affiliations:** ^1^ Second Department of Internal Medicine University of Toyama Toyama Japan

**Keywords:** echocardiography, speckle‐tracking echocardiography, strain

To the Editor,

A deep association between cardiac function, particularly global longitudinal strain (GLS) and COVID‐19 is receiving great concern. Khani et al.^1^ demonstrated that both right and left GLS were significant prognostic predictors in patients hospitalized to treat COVID‐19. Several concerns have been raised.

In their study, both right and left GLS were correlated with various other echocardiography parameters, respectively.^1^ They used only hypertension to adjust in the multivariate analyses. Other potential confounders including echocardiography parameters might exist. Also, the assessment to investigate the superiority of GLS to other echocardiography parameters would be of interest.

Baseline lower GLS was associated with mortality following COVID‐19 infection,^1^ but its detailed mechanism remains uncertain. Data on the cause of death should more clarify the association between GLS and mortality. Were most death related to cardiovascular diseases?

The authors measured GLS on admission.^1^ Some COVID‐19 survivors seem to have a persistent myocardial injury, which was assessed by decreased left GLS and elevated high‐sensitivity troponin levels.^2^ Prognostic impact of such a persistent myocardial injury remains the next concern.

## FUNDING INFORMATION

Teruhiko Imamura receives grant support from JSPS KAKENHI: JP20K17143.
